# Can Magnoflorine Improve Memory? Immunohistochemical Studies on Parvalbumin Immunoreactive Neurons and Fibers of Mice Hippocampus

**DOI:** 10.3390/nu17010137

**Published:** 2024-12-31

**Authors:** Radosław Szalak, Małgorzata Komar, Edyta Kowalczuk-Vasilev, Marta Kruk-Slomka, Justyna Zagórska, Marcin B. Arciszewski, Marcin Dziedzic, Wojciech Koch, Wirginia Kukula-Koch

**Affiliations:** 1 Department of Animal Anatomy and Histology, Faculty of Veterinary Medicine, University of Life Sciences, 12 Akademicka St., 20-950 Lublin, Poland; malgorzata.matysek@up.lublin.pl (M.K.); mb.arciszewski@wp.pl (M.B.A.); 2 Institute of Animal Nutrition and Bromatology, Faculty of Animal Science and Bioeconomy, University of Life Sciences, 13 Akademicka St., 20-950 Lublin, Poland; edyta.kowalczuk@up.lublin.pl; 3 Department of Pharmacology and Pharmacodynamics, Medical University of Lublin, 4a Chodzki Street, 20-093 Lublin, Poland; marta.kruk-slomka@umlub.pl; 4 Department of Food and Nutrition, Medical University of Lublin, 4a Chodzki St., 20-093 Lublin, Poland; justyna.zagorska@umlub.pl (J.Z.); kochw@interia.pl (W.K.); 5 Department of Laboratory Diagnostics, Medical University of Lublin, 1 Chodźki St., 20-093 Lublin, Poland; marcin8303@gmail.com; 6 Department of Pharmacognosy with Medicinal Plant Garden, Medical University in Lublin, 1 Chodzki St., 20-093 Lublin, Poland

**Keywords:** magnoflorine, parvalbumin, hippocampus, learning and memory, dementia, chromatography

## Abstract

**Background/Objectives**: We assessed the influence of long-term injection of magnoflorine (MAG) on memory acquisition in mice for the first time. **Methods**: This isoquinoline alkaloid that belongs to the aporphines was isolated from the roots of *Berberis vulgaris* by centrifugal partition chromatography (CPC) using a biphasic solvent system composed of chloroform: methanol: water in the ratio 4:3:3 (*v*/*v*/*v*) with 20 mM of hydrochloric acid and triethylamine, within 64 min. **Results**: Our results indicated that long-term injection of MAG 20 mg/kg dose improve the long-term memory acquisition in mice that were evaluated in the passive avoidance (PA) test with no toxicity records. The analysis of brain lysates and animal plasma by HPLC-ESI-QTOF-MS/MS showed the ability of the compound to cross the blood–brain barrier, and an elevated level of phosphatidylcholine PC (14:1(9Z)/14:1(9Z)) with the molecular formula of C_36_H_69_NO_8_P was observed in both treated groups with 10 mg/kg and 20 mg/kg MAG in comparison to the control group. **Conclusions**: This phenomenon may explain MAG’s cognition-enhancing properties as the PC may induce the synthesis and strengthening of neuronal cells. Also, the 7-day-long administration of MAG at 10 mg/kg and 20 mg/kg increased the mean number of parvalbumin (PV)-IR neurons in the hippocampus. Statistically, the largest PV-IR neurons were observed at the 20 mg/kg dose, which may indicate a potential effect of MAG on Ca^2+^ metabolism. However, no statistical differences were observed in the mean number of PV-IR nerve fibers in both doses of MAG, regardless of the hippocampal fields. This positive effect of MAG on hippocampal neurons provides further support for the neuroprotective effect of this alkaloid.

## 1. Introduction

Magnoflorine (MAG) is a vital isoquinoline alkaloid bearing an aporphine ring distributed in several plants representing the following botanical families: Ranunculaceae, Papaveraceae, Berberidaceae, and others. The representatives of the Berberidaceae botanical family have been widely consumed around the world and, particularly, in the Middle East as fruits rich in polyphenols with their characteristic acidic taste matching with meat-containing dishes and desserts or in the form of teas or dietary supplements from the powdered root or root bark [1,2,3]. Recent years have brought much interest in isolating single components from barberry shrubs, among them MAG for whom various biological properties have been proven in both in vitro and in vivo tests. MAG exhibited antiviral, hypotensive, antimicrobial, anticancer, and antiprotozoal activities [4,5]. Among the effects attributed to MAG, its inhibitory impact on acetylcholinesterase enzyme activity is worth noting. The alkaloid was proven to cross the blood–brain barrier and to induce central action, which includes its impact on memory acquisition and consolidation [6,7] Previous studies, also of the authors, showed that MAG was able to enhance memory of laboratory mice when administered intraperitoneally with a single injection [7]. Based on these initial results, its impact on the organism of a mouse, when delivered long-term, is of high interest, especially these days when we observe an increasing number of dementia cases in the elderly. Alzheimer’s disease (AD), dementia, or Parkinson’s disease (PD) are all emerging in the global society at a great tempo. The above units and other so-called ‘civilization diseases’ with a background directed to inflammation become a burden today [8]. That is why it is of the highest importance to fight for a prolongation of comfort and healthy life by searching for novel molecules, plant extracts, or physical activity tips that could enhance the brain’s activity despite an increasing life span.

In the neurodegenerative processes, a high, even toxic increase in intracellular calcium ions is observed. The calcium ions (Ca^2+^) are considered secondary intracellular transmitters/information messengers. They occur in the cell at low concentrations, stimulating many cellular processes, i.e., secretion of hormones and neurotransmitters, cell proliferation, gene expression, and neuronal signaling [9]. After cell stimulation, as a result of normal life processes or disturbance of natural regulatory functions, the level of Ca^2+^ increases almost tenfold, and the inside of the cell is “flooded” with calcium ions, causing destruction and even cell death (apoptosis). Thus, at high concentrations, calcium ions are cytotoxic [10]. For this reason, the level of intracellular Ca^2+^ must be constantly regulated to maintain the proper balance between calcium-dependent cell function and calcium-dependent cell death [11]. Various mechanisms are used to regulate the level of intracellular Ca^2+,^ i.e., pumps, ion channels, transporters, and calcium-binding proteins (CaBPs) [12,13]. CaBPs can quickly buffer incoming calcium, allowing neurons to reach homeostasis [14]. Parvalbumin (PV), one of the CaBPs present in inhibitory GABAergic neurons, is a high-affinity Ca^2+^ binding protein that, as a buffer, plays a vital role in regulating Ca^2+^ flow [15]. PV is thought to control calcium-dependent metabolic processes in GABAergic neurons. In addition, it participates in synaptic plasticity and affects the excitability of interneurons in the hippocampus by regulating other GABAergic neurons.

The hippocampus, a limbic system structure deeply embedded in the brain’s medial temporal lobe, is primarily responsible for long-term and spatial memory [16]. Intensive neurogenesis processes occur in it, regardless of the maturity of the organism. The hippocampus, on the one hand, is characterized by a high plasticity; on the other hand, it shows an increased vulnerability to neurodegeneration. The impairment of the hippocampus can lead to memory and cognitive decline [17,18].

The work presented here aims to investigate the effects of MAG on intracellular calcium ion levels by determining the number and size of immunoreactive PV neurons and nerve fibers (PV-IR). In addition, the molecular mechanisms are to be complemented by in vivo behavioral studies in mice to better observe the overall effect of the compound on the living organism, as well as the results of the HPLC-MS analysis of brain lysates, which determine the changes in the metabolism of mice after MAG administration. The study is made possible by using centrifugal partition chromatography, a hydrostatic type of countercurrent chromatography whose role was to efficiently obtain a sufficient amount of the high-purity alkaloid for biological studies.

## 2. Materials and Methods

### 2.1. Animals

The experiments were conducted on naive male Swiss mice (Experimental Medicine Center, Lublin, Poland) weighing 20–30 g, 4 weeks of age. Mice were housed in groups of 10 mice/home cages (38 × 22 × 18 cm^3^) made of white Plexiglas. The animals were maintained under standard laboratory conditions (12 h light/dark cycle, room temperature 21 ± 1 °C, and relative humidity of 55 ± 10%) with free access to tap water and laboratory chow (Agropol, Motycz, Poland) in their home cages and adapted to the laboratory conditions for one week. The total number of mice in the experiment was 22. Each experimental group consisted of 7–8 animals. The number of animals was selected to sustain proper statistical analysis of results. All behavioral experiments were performed between 8:00 and 15:00 on animals assessed visually to be in good health condition and putting on weight correctly, similar to other animals.

All studies were carried out according to the ARRIVE guidelines to improve the reporting of animal research and improve the quality of the studies and were conducted by the National Institute of Health Guidelines for the Care and Use of Laboratory Animals and with the European Community Council Directive for the Care and Use of Laboratory Animals of 22 September 2010 (2010/63/EU). Furthermore, on 19 June 2015, we obtained the agreement to conduct the aforementioned studies (number: 33/2015) from the Local Ethical Committee for Animal Experiments in Lublin.. After all, all gathered animals were used for the study. No randomization technique or blinding of the study was implemented. The experiments were performed incorporating the principles of substitution, limitations, and improvements (3R).

### 2.2. Drugs

MAG (10 mg/kg and 20 mg/kg b.w.) from the methanolic extract of *Berberis vulgaris* root was purified according to the previously described protocol [19] thanks to the application of centrifugal partition chromatography (CPC) as reported in a previous paper by the authors. The details of the purification that was based on the application of a biphasic solvent system composed of CHCl_3_: MeOH: H_2_O (4:3:3 *v/v*/*v*) with 20 mM of triethylamine and hydrochloric acid to the lower and upper phases, respectively, are presented in the Supplementary Materials attached to the manuscript.

For the behavioral assay, MAG was transferred to a mortar and dissolved in saline. All compounds were administered intraperitoneally (i.p.) at 10 mL/kg. Fresh drug solutions were prepared on each day of experimentation. Control groups received saline injections at the same volume and by the same route of administration.

### 2.3. Experimental Procedures

PA test apparatus:

The passive avoidance (PA) task was used in the study to measure the memory-related responses. For this purpose, an apparatus composed of an acrylic box with two compartments was used, one of which was a lighted one (10 × 13 × 15 cm) and the other a darkened one (25 × 20 × 15 cm). Fluorescent light (8W) was used to illuminate the lighted chamber. The dark chamber was equipped with an electric grid floor. During the experiment, the entrance of animals to a dark compartment was punished with an electric foot shock (0.2 mA for 2 s).

PA test experimental procedures:

First, a pretest was performed during which the animals were individually placed in a lighted compartment. They were allowed to explore the lighted box for 30 s. Later, the guillotine door was opened and the mice were able to enter the dark compartment. The time necessary for the animals to come from the lighted to the darkened box was measured and named the latency time 1 (TL1). As soon as an animal entered the darkened part of the box, the guillotine doors were closed and a 2-s-long electric foot shock (0.2 A) was applied to the animal through a metal grid floor. In the case the animal did not enter the darkened compartment within 300 s, it was placed into this dark box, the door was closed, and an electric foot shock was delivered to the animal, which was not the case in our study. In this case, the TL1 value would be noted as 300 s.

After 24 h the same animals were again individually placed in the PA apparatus in the lighted compartment to measure the memory retention. Again, after the adaptation of 30 s in the safe compartment, the guillotine door was raised and the time needed for an animal to enter the darkened compartments was measured and noted as TL2. This time, no foot shock was applied in the darkened part of the apparatus. The test lasted for 300 s. If the animal did not enter the dark compartment within this time, the test was stopped, with the result of TL2 equal to 300 s.

The changes in PA performance were presented as a latency index (LI) that was calculated as the following ratio:

- LI = TL2 − TL1/TL1
- TL1—the time taken to enter the darkened part of the PA apparatus during the training; TL2—the time necessary to re-enter the darkened box during the retention session [20,21].

The LI expressed the differences between the latencies within the retention and training stages and was determined for every animal in the tested group.

### 2.4. Treatment

MAG (10 mg/kg and 20 mg/kg) or saline for the control group was administeredlong-term i.p. for 7 days. On the 8th day, MAG (10 mg/kg or 20 mg/kg) or saline was administered i.p. Fifteen minutes after the last injection, a pretest (training) was conducted; whereas 24 h later, the behavioral test (retention) was performed.

### 2.5. Immunohistochemistry and Antibodies

One hour after the administration of the drug, the animals were euthanized (decapitated) and the collected brains were immediately stored in a 10% solution of buffered formalin (at pH = 7). After 12 h of storage at the temperature of 4 °C, the organs were gradually dehydrated with increasing concentrations of ethanol. As described previously [19], the brains were finally embedded in paraffin blocks and cut with a microtome into 5 μm thick sections (Microm HM 360, Microm, Walldorf, Germany). At the following stage of the analysis, the obtained brain sections were placed on the adhesive glass slides (Superfrost Plus, Thermo Scientific, Braunschweig, Germany) and used for further immunohistochemical staining with a peroxidase-antiperoxidase method.

For this purpose, the brain sections were first washed three times, 15 min each, in xylene in order to remove paraffin. Later, the samples were hydrated by sequential incubation in ethanol (in a graded series) and, in the end, washed using distilled water. In the following step, the tested samples were transferred to citrate buffer (pH = 6) and heated 3 times for 7 min to 97 °C using a microwave (800 W) in order to retrieve antigens. Then, the selected brain sections were outlined using a hydrophobic marker (ImmEdge^TM^ Hydrophobic Barrier Pen, Vector Laboratories, Burlingame, CA, USA). To inhibit the endogenous peroxidase activity, the analyzed sections were cooled down and later washed using the 3% solution of hydrogen peroxidase for the following 20 min. Then, the brain slices were flushed two times using the PBS solution at a pH of 7.4 (each time for 15 min) and subjected to the following incubation in 2.5% normal goat serum (ImPRESS^TM^, MP-7451, Vector Labs, Burlingame, CA, USA) at RT for the next 20 min. Then, for the following 24 h, the samples were incubated with primary monoclonal rabbit antibodies raised against PV (1:2000; SWANT, PV25, Fribourg, Switzerland) at 4 °C. After this procedure, the slides were washed two times for 15 min using a washing buffer and covered for 1 h with the anti-rabbit immunoglobulin (ImPRESS^TM^, MP-7451 Vector Labs, Burlingame, CA, USA). The 3,3′-diaminobenzidine chromogen was used to visualize primary antisera (ImmPACT^TM^ DAB, SK-4105, Vector Labs, Burlingame, CA, USA). For the following 20 min, the counterstaining with Mayer’s hematoxylin was performed. After this procedure, the brain sections were rinsed with distilled water and the sections were again dehydrated using the gradually increasing ethanol concentrations. Later, the samples were cleared using xylene, mounted in DPX (Sigma-Aldrich, St. Louis, MO, USA), and cover-slipped.

To test the antibodies’ specificity, two different procedures were applied. At the beginning, the negative control samples that were not exposed to the influence of primary antibodies (which were replaced with non-immunoreactive sera or omitted) were stained. Then, the procedure was a pre-absorption experiment in which primary antibodies were mixed with an excess of target synthetic protein before incubation. As a result, we did not observe any positive immunoreactions in these control procedures.

The obtained slides were assessed under a light microscope (Axiolab, Zeiss, Jena, Germany) and the images were recorded by a digital camera (C11440-36U, Hamamatsu Photonics, Shizuoka, Japan) that was connected to a standard PC with an installed CellˆM 2.3 image analysis software (version 13.1, Olympus cellSens Standard, Olympus Corporation, Tokyo, Japan). The images were acquired using a 20× magnification objective with 1024 × 1024 pixels resolution. During the study, ca. 25–30 immunostained sections for PV were examined from each animal**.** Explanation: in this work, the term “neuron” refers to the body of the neuron, the perikaryon. Therefore, the average number of perikaryons, their average size, and the mean number of nervous fibers were determined. The average numbers of PV-IR neurons presented in this study were obtained by counting and analyzing no less than a hundred neurons from each area: the CA1–CA3 fields and the DG of the hippocampus of the experimental groups and the control groups. Moreover, all PV-IR neuron measurements in the DG and all hippocampal fields were prepared in three cross-sections—horizontal, vertical, and diagonal—to precisely calculate the average neuron size. The performed morphometric analysis of the analyzed parvalbumin-immunoreactive (PV-IR) neurons was achieved using the CellˆM 2.3 software. Additionally, in the DG of the hippocampus and in every area of CA1–CA3 of the tested and control groups, the average number of PV-IR nerve fibers from at least a hundred neurons with and without PV-IR reactions was determined using the CellˆM 2.3 software (Olympus cellSens Standard). To increase the accuracy of the performed experiments, at least two independent observers were engaged in the quantification studies and the obtained results were averaged.

### 2.6. HPLC-ESI-QTOF-MS/MS Analysis of Brain Lysates

Three brains from each tested group were homogenized using 0.5 mL of acetonitrile water (70:30 *v/v*). The homogenization was performed in plastic 2 mL volumed Eppendorff tubes that were inserted into a beaker filled with crushed ice to prevent heating. The plasma samples—3 samples from each group—were vortexed with equal volumes of the same mixture of acetonitrile: water (70:30 *v*/*v*). All samples were centrifuged at 12,500 rpm at 5 °C for 30 min and the supernatants were frozen at −80 °C overnight. The next day, after defrosting, the samples were centrifuged again. The supernatants were filtered through nylon syringe filters with a pore diameter of 0.1 µm into autosampler vials with inserts, and they were subjected to HPLC-MS analysis. A similar procedure was applied to the samples from the animals’ control group. The chromatographic analysis was conducted on an RP-18 Zorbax Eclipse Plus column (150 mm × 2.1 mm, 3.1 µm pore diameter) (Agilent Technologies, Santa Clara, CA, USA) in a gradient of acetonitrile with the addition of 0.1% formic acid (A) in water with 0.1% formic acid, according to the following program: 0 min—10% A, 10–12 min—40% A, 17 min—95% A, and 20-35 min—10% A. The flow rate was set at 0.2 mL/min, the temperature at 20 °C, and the analysis time at 35 min. The mass spectrometer (6500 series) was attached to an HPLC chromatograph (1200 Series) with a degasser, a binary pump, an autosampler, and a UV detector and operated in the positive ion mode within the *m*/*z* range of 40–1000 Da, with gas flow and sheath gas temperatures of 250 and 300 °C, respectively, gas flows of 12 L/min, capillary voltage of 3000 V, fragmentor voltage of 110 V, skimmer voltage of 65 V, and collision energies of 10 and 20 V. The quantitative analysis was performed in the MS mode based on three injections and, for comparison, with a five-point calibration curve drawn for the standard compound. In contrast, the identification of MAG in the sample was achieved in the MS/MS mode by analyzing the fragmentation pattern of the compound with high *m*/*z* weight accuracy, as described previously in the manuscript by the authors [19]. The data were handled by the Agilent Technologies program, Mass Hunter (version B.10.00).

Detailed chemometric analyses were conducted based on the mass chromatograms and mass data in the Mass Profiler Professional Program (version 15.1, Agilent Technologies). First, the list of molecular features was extracted from the recorded files in the Profinder program (v.10.0.02, Agilent Technologies) based on the intensity and retention time and used for the direct comparisons of the injections from the tested animals—both control and treated groups. The collected data were subsequently filtered in consideration with the following criteria: the mass tolerance of 10 ppm, the peak area of at least 2% of the most intensive *m*/*z* signal, and the retention time variation of 0.5 min. The extracted data were then analyzed in the Mass Profiler Professional Program to determine the differences between the samples coming from the treated and control groups. For this purpose, the Kegg pathway database was used in the Pathway Analysis module of the Mass Profiler Professional Program to search for the actual impact of MAG on the variations in the biochemical markers in the brain. The analysis was performed based on the list of 3796 identified molecular features in the positive ion mode assigned to mice metabolites using the commonly available mass library—Metlin. A one-way ANOVA with asymptotic *p*-value computation and Benjamini–Hochberg multiple testing correction (*p* < 0.001) were used for the statistical analyses. Also, the KEGG pathway database (the Kyoto Encyclopedia of Genes and Genomes, Mass Profiler Professional, Santa Clara, CA, USA) was considered when analyzing the recorded mass data from the injections of biological material of the tested groups administered with 10 mg/kg and 20 mg/kg b.w. of MAG and a control group, to trace the molecular changes introduced by MAG treatment.

### 2.7. Anti-Inflammatory Properties

Using the aliquots of 50 µL of plasma from all tested groups (10 mg/kg and 20 mg/kg b.w. i.p. of MAG and a saline group), the levels of IL-6 and TNF-alpha were determined to exclude eventual toxic reactions provided by the alkaloid. The tests were performed using the commercially available enzyme-linked immunosorbent assays, namely, a murine IL-6 and TNF alpha kits by Diaclone Immunology Products & Service (Besancon, France) according to the methodology suggested by the producer.

### 2.8. Statistical Analysis

The statistical analysis of results was conducted using a one-way or two-way analysis of variance (ANOVA) for the factors of pretreatment, treatment, and pretreatment/treatment interactions. When appropriate, the post hoc comparison of means was carried out using Tukey’s test (for one-way and two-way ANOVA) for multiple comparisons.

The data were considered statistically significant at a confidence limit of *p* < 0.05. The ANOVA with Tukey’s post hoc test was performed using GraphPad Prism version 7 for Windows (GraphPad Software, San Diego, California, USA, www.graphpad.com, accessed on 2 November 2024).

Other acquired data were processed with Statistica software ver. 13.1 (StatSoft, Kraków, Poland). The Kolmogorov-Smirnov test was used to assess the normality of the obtained results, whereas Levene’s homogeneity of variance test was used to study the equality of variances. Additionally, a one-way ANOVA test was used to assess the influence of MAG treatment (irrespective of its dose) in comparison to the non-treated control group. Also, a two-way ANOVA test was applied to study the effect of the MAG dose (0 mg, 10 mg, 20 mg) and the analyzed field of the hippocampus (CA1, CA2, CA3, and DG), as well as the interaction of both factors on the number of PV-IR neurons, their size, and PV-IR nerve fibers. To estimate the possible impact of neuronal size, a 3-way ANOVA was performed, which included a third possible factor: vertical, horizontal, and diagonal. Post hoc Tukey’s analysis was performed to estimate the significant differences between the groups at a significance level of *p* ≤ 0.05 and *p* ≤ 0.01. Additionally, to assess the strength of the relationship between the dose of MAG and the number of PV-IR neurons and nerve fibers, the Pearson correlation coefficients were estimated at a significance level of *p* ≤ 0.05.

## 3. Results

### 3.1. The Influence of Chronic Administration of MAG on Memory Acquisition in the PA Test in Mice

The table below shows the latency time (TL in s) during which the animals entered the dark room in the first (TL1, training) and second (TL2, retention) trial of the PA test for each group (Table 1). As proven in the study in the first round of the experiment, all animals entered the dark compartment within 300 s.

Based on the results of TL1 and TL2, the LI described in the methodology section was calculated. The statistical analysis included the analysis of LI values for individual groups of mice.

**Figure 1:**
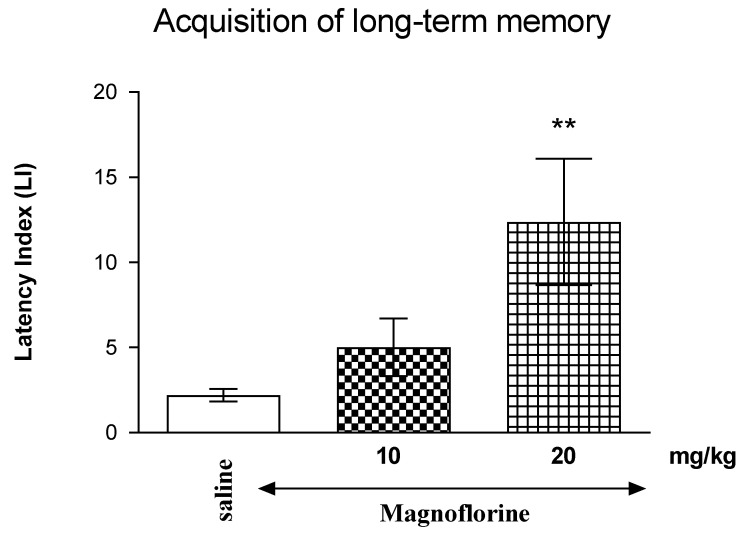
Effects of long-term magnoflorine (MAG) injection on the latency index (LI) values determined in the passive avoidance test on memory acquisition in mice. MAG (10 mg/kg or 20 mg/kg, i.p.) or saline was administered for 7th days. Afterward, on the 8th day and 15 min after the last injection, the trial was conducted and the TL1 times were determined. The experiment was repeated 24 h later and the TL2 was determined; n = 7–8; the means ± SEM; ** *p* < 0.01 vs. saline-treated control group; Tukey’s test.

A one-way ANOVA revealed that long-term i.p. administration of MAG (10 mg/kg and 20 mg/kg) had a statistically significant effect on LI values for memory acquisition [F(2.21) = 5438; *p* = 0.0136]. Indeed, long-term treatment with MAG at the dose of 20 mg/kg significantly increased LI values in the PA test in mice compared to those in the saline-treated control group (*p* < 0.01), a post hoc Tukey’s test (Figure 1), indicating that long-term administration of MAG at this used dose improved the acquisition of memory and learning in mice in the PA test.

### 3.2. Immunohistochemistry

PV immunoreactivity in CA1–CA3 fields of the hippocampus and DG.

**Figure 2:**
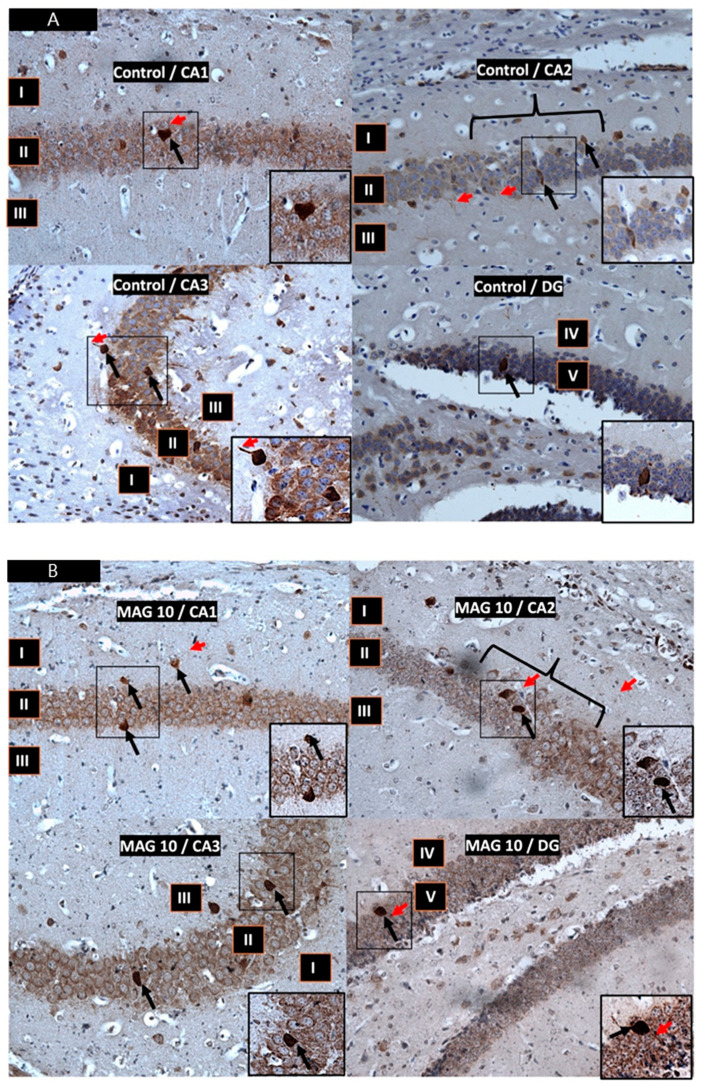

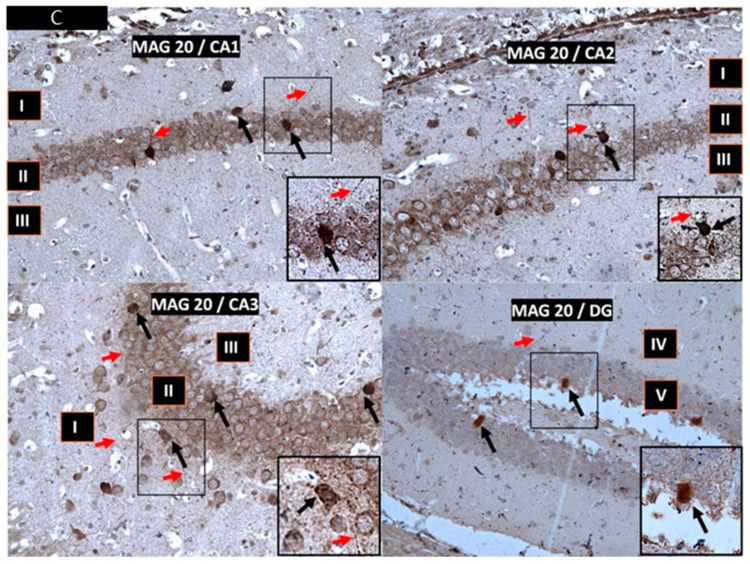
(**A**). Control group. Immunoreactivity of PV-IR neurons in the fields CA1–CA3 and the DG of the mouse hippocampus were observed. (**B**). Group MAG10. Immunoreactivity of PV-IR neurons in the fields CA1–CA3 and the DG of the mouse hippocampus were observed. (**C**). Group MAG20. Immunoreactivity of PV-IR neurons in the fields CA1–CA3 and the DG of the mouse hippocampus were observed. I—the marginal layer; II—the pyramidal layer; III—the multiform layer; IV—the molecular layer; V—the granular layer. The arrows indicate PV-IR neurons (black/long arrow) and PV-IR nerve fibers (red/short arrow) in the hippocampus. Magnification 20×.

The hippocampus, also known as Ammon’s horn (CA), is a small brain structure located in the posterior part of the temporal lobe, part of the limbic system. The hippocampus is divided into three parts called fields/segments (CA1–CA3) and dentate gyrus (DG). Functionally, the hippocampus consists of a dorsal part, which mainly performs cognitive and spatial functions, and a ventral part, which is involved in emotions and stress. In this study, the dorsal hippocampus was examined. In all study groups, multiform (oval, round, triangular, and fusiform) immunoreactivity neurons of PV (PV-IR) were observed, unevenly distributed in marginal and pyramidal layers of the mouse hippocampal fields and in the granular layer of the DG. Neurons were characterized by cytoplasmatic and nuclear reactions although the cytoplasm was more intensely stained (Figure 2A–C). The immunoreactivity to parvalbumin (PV) was also proven in the case of the nerve fibers of both the DG and all tested hippocampal fields: CA1, CA2, and CA3. 

### 3.3. Results of the Quantitative Study

**Figure 3:**
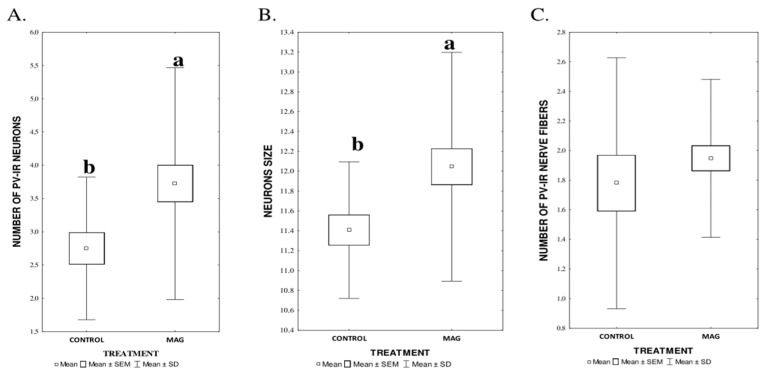
(**A**–**C**). The average number of PV-IR neurons (**A**), their size (**B**), and the average number of PV-IR fibers (**C**) in the mouse hippocampus treated with magnoflorine (MAG) irrespectively of its dose. The data are expressed as means ± SEM (standard error of the mean; box) and standard deviation (whiskers); a, b—different letters indicate significant differences between the experimental groups at *p* ≤ 0.05 *n* = 5.

MAG supplementation, irrespectively of the dose, significantly influenced both the average number of PV-IR neurons (*p* = 0.025) in all fields of the mouse hippocampus and the DG, compared to the control group and, also, the average size of neurons (*p* = 0.026). At the same time, there was no significant difference between the control group and the groups supplemented with MAG in the average number of PV-IR nerve fibers (*p* = 0.35) (Figure 3A–C).

**Figure 4:**
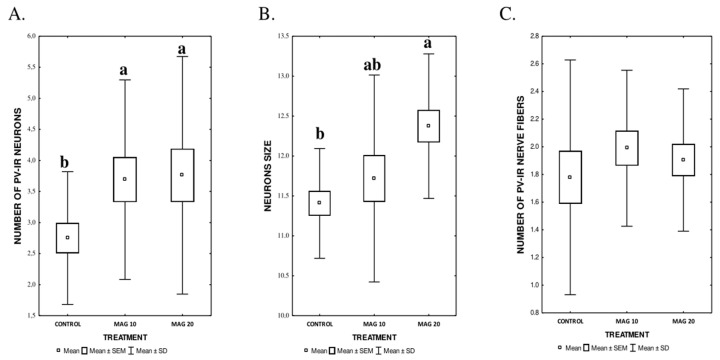
**A**–**C.** The graphs show the average number of PV-IR neurons (**A**), their size (**B**), and the average number of PV-IR fibers (**C**) present in the mouse hippocampus calculated in the magnoflorine-treated (MAG) groups, depending on the dose. The presented data are shown as means ± SEM (standard error of the mean; box) and standard deviation (whiskers); a, b—different letters indicate significant differences between the experimental groups at *p* ≤ 0.0001, *n = 5*.

The dose of MAG significantly affected the mean number and mean size of PV-IR neurons (*p* = 0.0001). The highest average number of PV-IR neurons (*p* ≤ 0.0001), with the highest size at the same time (*p* = 0.0008), was found in the MAG20 group. In the MAG10 group, a significantly higher average number of PV-IR neurons was found compared to the control group. However, the size of neurons in the MAG10 group did not differ significantly from the control group (Figure 4A,B). There were no statistically significant differences between both groups supplemented with MAG (MAG10 and MAG20), taking into account the average number of neurons and their average size (Figure 4A,B). In contrast, depending on the MAG dose, no significant differences in the mean number of PV-IR nerve fibers were found (Figure 4C).

**Figure 5:**
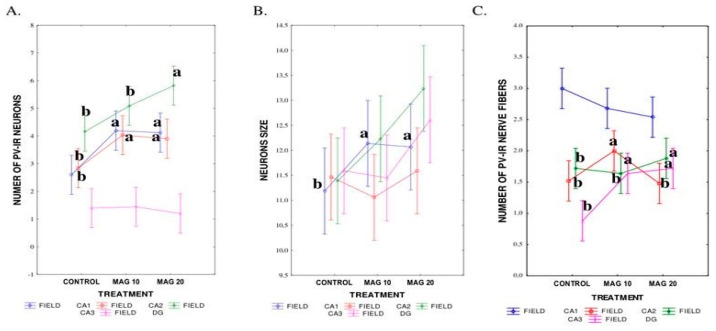
(**A**–**C**). Differences in the average number of PV-IR neurons (**A**), neuron size (**B**), and PV-IR nerve fibers (**C**), depending on the dose of magnoflorine (MAG) in the analyzed fields. The data are expressed as means ± SEM (standard error of the mean; box) and standard deviation (whiskers); a, b—different letters indicate significant differences between the experimental groups at *p* ≤ 0.0001. *n =* 5.

The MAG dose exerted a statistically significant effect on the mean number of PV-IR neurons in each analyzed hippocampal field (CA1, CA2, CA3). In the CA1 field, statistically significant differences were observed in the average number of PV-IR neurons in the MAG10 and the MAG20 groups compared to the control group. Similar results were obtained in the CA2 hippocampal field where the supplementation of MAG caused an increase in the mean number of PV-IR neurons. In turn, in the CA3 hippocampal field, statistically significant differences in the mean number of PV-IR neurons were found only in the MAG20 group compared to the MAG10 and the control groups (Figure 5A).

However, in the DG, no significant differences in the mean number of PV-IR neurons were observed between the treatments (Figure 5A).

Considering the average size of PV-IR neurons, significant differences occurred only in the CA1 field, compared to the CA2 and CA3 hippocampal fields and the DG (Figure 5B). The statistically significant differences in the mean size of PV-IR neurons in the CA1 field were observed between both groups supplemented with MAG and the control one (Figure 5B). The average size of neurons was measured on the three axes: vertical, horizontal, and diagonal; after each measurement, the average size of the neuron was estimated.

Although, in general, the addition of MAG did not significantly affect the total number of nerve fibers, analysis of each field individually allowed us to show the differences between the groups and revealed the different impacts of the treatment on nerve fibers in the analyzed fields.

Significant differences were observed in the CA2 field of the MAG10 group where the addition of MAG caused an increase in the mean number of PV-IR nerve fibers compared to the MAG20 group and the control group. Likewise, in the DG, the addition of MAG caused an increase in the mean number of PV-IR nerve fibers, but in comparison to the CA2 field, this increase was observed in both the MAG groups (10 mg/kg and 20 mg/kg) compared to the control group. In the CA3 hippocampal field, the supplementation of MAG also significantly affected the mean number of PV-IR nerve fibers, but only in the MAG20 groups. In the CA1 field, there were no statistically significant differences in the average number of PV-IR nerve fibers depending on the MAG dose (Figure 5C).

All things considered, the mean number of PV-IR neurons and PV-IR nerve fibers were significantly affected by both the MAG supplement (*p* < 0.0000) and the analyzed field (*p* < 0.0000). The interaction of both experimental factors significantly affected the mean number of PV-IR neurons (*p* = 0.02) and the mean number of PV-IR nerve fibers (*p* = 0.003). The addition of MAG also affected the size of PV-IR neurons (*p* = 0.037), with no differences in the hippocampal field.

Pearson’s correlation coefficient analysis showed that long-term MAG treatment had a powerful positive impact (r = 0.99) on its concentration found in the hippocampus. Furthermore, the number of neurons and their size were strongly correlated with the applied dose (r~0.7). These observations were also confirmed by the strong correlation between the actual MAG concentration in the hippocampus and these analyzed traits. The strongest statistically significant positive effect on the average number of PV-IR neurons was stated in the CA1 and CA3 fields of the hippocampus. At the same time, no significant relation was expressed between the average number of PV-IR nerve fibers and the increasing dose of MAG. The analysis confirmed that the accumulation of MAG in hippocampus tissue has the most substantial significant impact on the CA1, CA2, and CA3 fields but not the DG (Table 2).

### 3.4. The Anti-Inflammatory Potential of MAG

**Figure 6:**
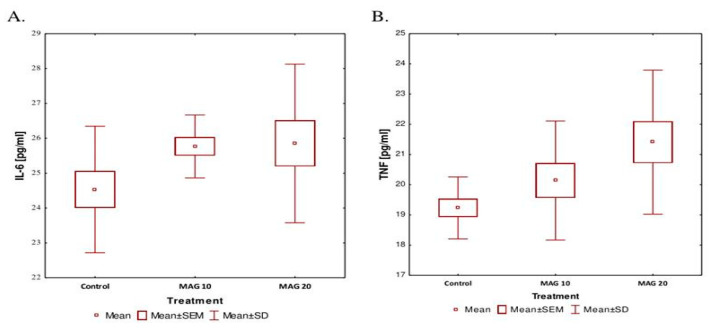
Levels of selected IL-6 (**A**) and TNFα (**B**) in the blood plasma of animals after long-term administration of magnoflorine (MAG).

The plasma samples of the tested groups of animals were treated with MAG at 10 mg/kg and 20 mg/kg b.w. i.p. for 7 consecutive days, and the control group receiving saline was studied for the interleukin IL-6 and TNF-alpha levels. The ELISA assays were performed to check whether the drug administration elevated the selected factors, which could indicate the toxic effects of MAG in a living organism. The study concluded that both treated groups showed no statistically significant differences in the levels of IL-6 (*p* = 0.201) and TNF-alpha (*p* = 0.076) in comparison with the control group injected with saline (Figure 6A,B).

### 3.5. HPLC-ESI-QTOF-MS-Based Quantitative Determination of Magnoflorine in the Mouse Brain and Plasma

**Figure 7:**
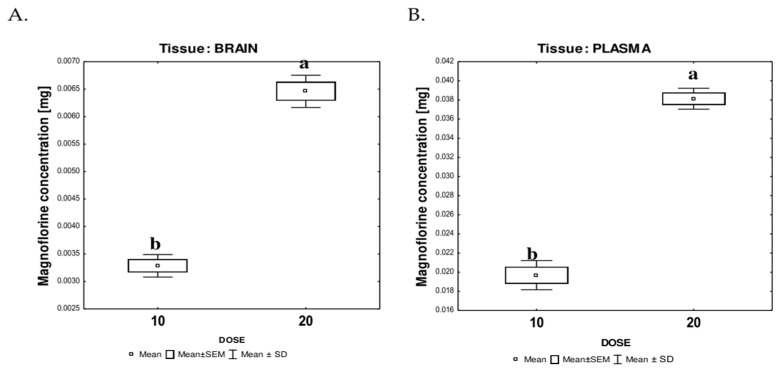
The concentration of MAG in brain lysates (**A**) and blood plasma (**B**) of experimental animals treated with different levels of MAG. The data are expressed as means ± SEM (standard error of the mean; box) and standard deviation (whiskers); a, b—different letters indicate significant differences between the experimental groups at *p* ≤ 0.0001. *n* = 5.

The performed HPLC-MS analysis provided valuable data that confirmed the presence of MAG in the analyzed biological material, that is, in the brain and plasma of the tested mice (Figure S1 in the Supplementary File). Figure 7 shows the results of the quantitative analysis of MAG, which were calculated based on the calibration curve equation recorded for the standard (Sigma Aldrich, St. Louis, MO, USA) y = 18120948982x + 5801179 (R^2^ = 0.9934).

As presented above, MAG was found to be present in brain structures. Even if its content was not high, it was sufficient to exhibit certain actions in the brain and influence the growth of neurons as proven by behavioral and immunohistochemical studies. The final calculated content of MAG in brain and plasma samples was data-dependent. The averaged measured content of MAG in the plasma was equal to 0.01969 ± 0.0015 mg/0.1 mL of plasma and 0.03814 ± 0.0011 mg/0.1 mL for 10 mg/kg and 20 mg/kg MAG, respectively.

In the case of brain samples, the analyses showed the total content of MAG per brain calculated as 0.003285 ± 0.00021 and 0.006461 ± 0.00029 mg/brain for 10 mg/kg and 20 mg/kg MAG, respectively. In both treated groups, the ratio of MAG in the plasma to brain ranged around 6:1.

In both types of biological material, the differences in MAG concentrations between the groups were significant (*p* = 0.0001 for the brain and *p* = 0.00007 for plasma).

The analysis of the injected homogenates and plasma samples in an untargeted metabolomics analysis provided additional data on the influence of MAG on the physiological status of the treated animals.

**Figure 8:**
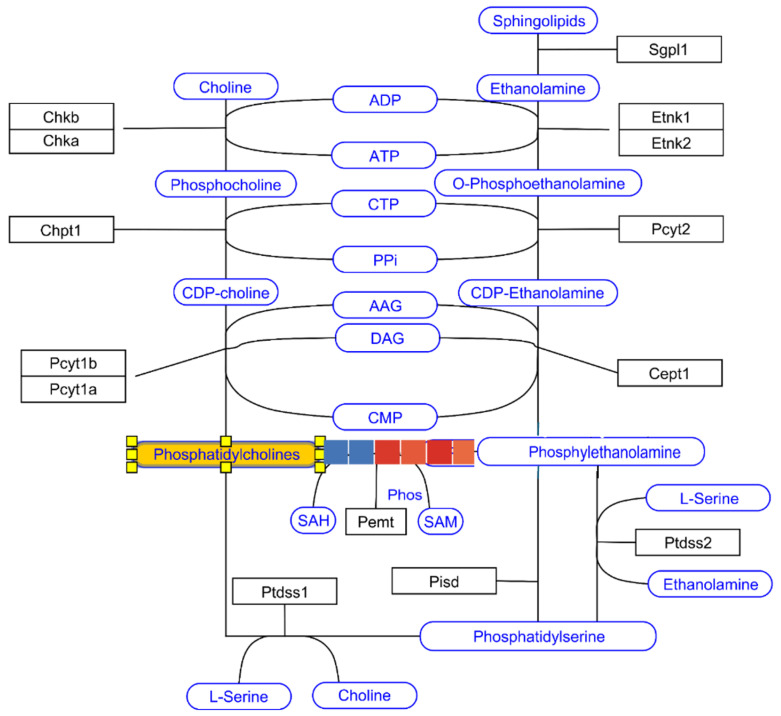
The impact of MAG on the Kennedy pathway indicating an upregulation in the phosphatydylcholine levels (C-control group, 10—10 mg/kg magnoflorine-treated group, 20—20 mg/kg magnoflorine-treated group).

Thanks to the application of the Mass Profiler Professional program coupled with the KEGG database and pathway analysis, visible changes in the content of phosphatidylcholine between the control group and the tested groups of animals were indicated (Figure 8, and Figure S2 in the Supplementary File) when analyzing plasma samples.

The interactions between the treatment with MAG and the content of phosphatidylcholines were indicated when analyzing the recorded mass chromatograms with the KEGG database. The content of phosphatidylcholine with the KEGG ID of c00157 named PC (14:1(9Z)/14:1(9Z)) and the molecular formula of C_36_H_69_NO_8_P were elevated in both treated groups with 10 mg/kg and 20 mg/kg MAG in comparison to the control group.

This observation may indicate that the alkaloid enhances the synthesis of cellular phosphatidylcholines, which may be promising for the synthesis and strengthening of cells’ functions in neurons [22].

## 4. Discussion

It is well known that in neurodegenerative diseases, nerve cell degeneration is caused by the deposition of pathological proteins, degenerative changes, or incorrect processing of neurotransmitters. During AD, one of the most well-known diseases involving memory disorders, there is an accumulation of a fragment of the β-amyloid protein, which, when tangled, disrupts the Ca^2+^ homeostasis, consequently leading to the disruption of calcium ion signaling pathways [10,23]. The proper functioning of neurons is primarily ensured by the appropriate concentration of calcium ions, regulated intracellularly by mitochondria, the endoplasmic reticulum, and parvalbumin as a cytosolic calcium-binding protein. Thus, there is an urgent clinical need to discover promising, effective drugs to protect brain neurons from damage.

As mentioned in the Introduction section, isoquinoline alkaloids are very important ingredients of medical significance that have been used in different traditional medicine canons. Among them, berberine, palmatine, berbamine, nuciferine, tetrandrine, and MAG were identified as metabolites with a wide range of pharmacological effects, which can be used in the treatment of many diseases, especially of the central nervous system, including the neurodegenerative disorders (AD, PD), cerebral ischemia, and nerve cell injury [24].

So far, MAG, after berberine, is another well-known isoquinoline alkaloid and an active plant compound distributed in barberry shrubs, *Coptis* or *Magnolia* species. This alkaloid exhibits various key pharmacological effects, including its immunomodulatory, anticancer, anti-inflammatory, and antioxidant action, as well as neuropharmacological properties [25,26].

In our previous study, we assessed the influence of the acute injection of MAG on the memory-related responses in the PA test. The improvement of long-term memory acquisition was observed at the doses of 20 mg/kg for MAG and 5 mg/kg for berberine. Additionally, both compounds attenuated memory impairment induced by scopolamine injection [7].

This study was performed to check the influence of MAG on this memory stage after long-term administration. In the described protocol, the influence of long-term (7 days) injection of MAG on memory-related responses in mice was assessed for the first time. To determine the impact of the compound on the cognitive functions of mice, the PA test was used similarly to the former study. As a result, the procognitive effect of MAG was confirmed. Also, an increase in the LI value after an injection of MAG, both 10 mg/kg and 20 mg/kg, in comparison to the saline control group was proven. Long-term injection of MAG (7 days) at the dose of 20 mg/kg statistically significantly improved long-term memory acquisition in mice in the PA test. In the case of a 10 mg/kg dose of MAG, no statistical significance was demonstrated, but the direction was indicated, showing the procognitive potential of MAG both after a dose of 10 mg/kg and 20 mg/kg.

In the context of our result, the most important seems to be the data concerning the influence of MAG on memory and learning processes and memory disturbances occurring in neurodegenerative diseases.

According to the previously published results, MAG ameliorated cognitive deficits in APP/PS1 mice in other behavioral tests, e.g., a water maze test and novel object recognition. Additionally, MAG reduced the neuronal apoptosis and conferred neuroprotection via inhibiting phosphorylated c-Jun N-terminal kinase (JNK) signaling pathway in the same AD model [27].

Another possible mechanism of MAG’s procognitive effect is the inhibition of cholinergic transmission and, thus, the influence of the compound on the acetylcholine level. Our previous study revealed that MAG has an anticholinesterase activity [19,28].

Even though the mechanism of action resembles the one of berberine, it is worth paying attention to its different structure. Contrary to berberine, which belongs to protoberberines, MAG is an apoporhine alkaloid, which may result in differences in toxicity. This information is of particular importance these days. Despite the common availability of berberine as a dietary supplement, there are some voices proving its hepatotoxic properties in in vivo studies [29]. In light of these findings, MAG with its similar mechanism of action, may become a substitute for berberine as it can be easily isolated from natural sources in high quantities, which has been proven in this study as well.

In this study, MAG did not interfere with locomotion in mice at the doses of 10 mg/kg and 20 mg/kg i.p. and was found to be less toxic or nontoxic to most cells and organs and well tolerated [7,25,30]. Also, the levels of IL-6 and TNF-alpha were not disrupted by the administration of MAG at both studied doses.

In the present work, we assessed the PV immunoreactivity to determine the effect of MAG on immunoreactive neurons of the mouse hippocampus as a specific memory and reasoning center. We found that regardless of dose, MAG supplementation significantly affected the mean number of PV-IR neurons in MAG groups compared to the control group. We obtained similar results in our previous studies [19] where short-term administration of MAG also resulted in an increase in the mean number of PV-IR neurons in experimental groups. The addition of MAG also significantly influenced the average number of PV-IR neurons depending on the hippocampal field. With long-term MAG administration, we recorded the greatest increase in the mean number of PV-IR neurons in the CA3 field in the MAG20 group, while with acute MAG administration, an increase in the mean number of PV-IR neurons was noted in all hippocampal fields, but the results obtained in this case differed depending on the field of the hippocampus and MAG’s dose—in the CA1 field and the DG, the largest significant increase in the average number of PV-IR neurons was recorded in MAG20 group, while in the CA2 and CA3 fields, it was in the MAG10 group.

In the present work, we also assessed the average size of PV-IR neurons. We observed that statistically larger PV-IR neurons are present in the group supplemented with MAG at a dose of 20 mg/kg, especially when compared to the MAG10 group and the control group. However, during a short administration of MAG, a statistically significant increase in the average size of neurons was observed in the MAG10 group, especially in the CA2 and CA3 fields of the hippocampus. However, considering the average number of PV-IR nerve fibers with chronic MAG administration, no statistically significant differences were observed between the control group and the experimental groups. We obtained similar results during the short administration of MAG [19].

The effect of MAG on the mean number and mean size of neurons may suggest its beneficial role in brain structures. MAG affects calcium homeostasis through its effect on PV-IR neurons and nerve fibers. Therefore, changes or disruptions in Ca^2+^ transport can severely affect intracellular calcium ion levels, causing neuronal cell dysfunction, ultimately leading to apoptosis.

The analysis of mass spectra from the control and tested groups let us notice marked differences in the levels of phosphatidylcholine peak. Elevated peak areas of the PC (14:1(9Z)/14:1(9Z)) compound with the molecular formula of C_36_H_69_NO_8_P characterized the 10 mg/kg and 20 mg/kg MAG treated groups contrary to the control group. As found in the scientific literature [22,31] phosphatydylcholine plays an important role in sustaining the differentiation and plasticity of neurons at the expense of the unspecified and astroglial precursors. Tests performed in neuronal stem cells underlined the role of glycerophospholipids connected with the proneurogenic effect. Also, for phosphatydylcholine, it has been proven that under the conditions of developed inflammation, it promotes the renewal mechanisms that can lead to the reparation of neuronal tissue and decrease the occurring functional and morphological deficit. This information is in line with a previous communication [32] that underlined that an increased phosphatydylcholine supplementation may be associated with the prevention of cognitive decline. Considering the information on phosphatydylcholine presented above, it is certain that a physiological increase of this metabolite may be crucial in the process of memory acquisition and regeneration from the inflammatory conditions that occur in dementia and brain injuries.

## 5. Conclusions

The results obtained in our experiments suggest that MAG has a positive effect on cognitive functions. We showed that chronic administration of MAG improved the acquisition of long-term memory in mice in the PA test. Chronic i.p. treatment with MAG (7 days) at a dose of 10 mg/kg caused an increase in the number of PV-IR neurons in mice, even if the results of the behavioral studies showed a statistically insignificant memory-enhancing tendency in comparison with the control group. For the behavioral tests, a higher tested dose of 20 mg/kg i.p. delivered unambiguous proofs and statistically significant results on the memory-enhancing properties of this alkaloid. Nevertheless, the molecular studies performed on both tested doses underline the occurrence of changes in the brain structure and an increased concentration of phosphatidylcholine (14:1(9Z)/14:1(9Z) compound with the molecular formula of C_36_H_69_NO_8_P) in the plasma of the treated groups in comparison with the control group in the HPLC-MS studies, which may promise the regenerative action toward neurons affected by the treatment of MAG. In light of the above information, it is worth considering the idea that this compound could be a promising new candidate for future use in the treatment of neurodegenerative and central nervous system diseases, including AD, especially when it is possible to recover high loads of the alkaloids from natural sources.

## Figures and Tables

**Table 1:** The influence of long-term magnoflorine (MAG) injection on the latency time (TL in s) during the memory acquisition trial using the PA test in mice. TL to enter the dark compartment was determined during traing and retention. MAG (10 mg/kg or 20 mg/kg, i.p.) or saline was administered for 7 days. On the 8th day, 15 min after the last injection, the first trial was conducted (TL1). The mice were retested 24 h later (TL2); n = 7–8.

	SALINE		MAGNOFLORINE 10 mg/kg		MAGNOFLORINE 20 mg/kg	
Number of mice	TL1	TL2	TL1	Tl2	TL1	TL2
1	32	91	98	300	25	300
2	84	227	30	274	10	27
3	90	300	37	146	173	300
4	73	300	73	300	7	217
5	129	281	72	300	18	300
6	127	300	15	40	21	300
7	34	181	20	300	17	259
8	22	61				

**Table 2:** Pearson’s correlation coefficients (r) between the dose of magnoflorine (MAG) and its concentration in the mouse hippocampus and its impact on PV-IR neurons and the number of PV-IR nerve fibers in different fields (CA1, CA2, and CA3) and the DG. Values of correlation coefficients in bold are statistically significant (*p* ≤ 0.05).

Parameter	PEARSON’S CORRELATION COEFFICIENTS	
	MAG Dose	MAG Concentration in Hippocampus
MAG concentration	0.99	
Number of PV-IR neurons		
Total	0.65	0.73
CA1 PV-IR neurons	0.52	0.61
CA2 PV-IR neurons	0.54	0.59
CA3 PV-IR neurons	0.62	0.59
DG PV-IR neurons	−0.35	−0.21
Neurons size		
Total	0.75	0.63
CA1 neurons size	0.53	0.63
CA2 neurons size	0.10	0.07
CA3 neurons size	0.68	0.61
DG neurons size	0.29	0.18
Number of PV-IR fibers		
Total	0.29	0.43
CA1 PV-IR nerve fibers	−0.43	−0.43
CA2 PV-IR nerve fibers	−0.05	0.21
CA3 PV-IR nerve fibers	0.29	0.14
DG PV-IR nerve fibers	0.64	0.70

## Data Availability

The data that support the findings of this study are available from the corresponding authors upon reasonable request.

## Supplementary Materials

The following supporting information can be downloaded at: https://www.mdpi.com/article/10.3390/nu17010137/s1, Figure S1: Extraction of plant material and isolation of MAG from the roots of Berberis vulgaris; Figure S2: The details of the chemometric analysis of the samples shown in the Kegg Pathways analysis and the details obtained for plasma sample of control and two treated groups with 10 mg/kg and 20 mg/ kg MAG1.
