# Supplementary material for: Can Magnoflorine Improve Memory? Immunohistochemical Studies on Parvalbumin Immunoreactive Neurons and Fibers of Mice Hippocampus
Source: Nutrients. 2024 Dec 31;17(1):137. doi: 10.3390/nu17010137 (PMC11722722; doi:10.3390/nu17010137)
Supplement: Supplementary file 1 [file nutrients-17-00137-s001.zip › nutrients-3370340-supplementary.pdf]

SUPPLEMENTARY MATERIAL

S1. Extraction of plant material and isolation of MAG from the roots of *Berberis vulgaris*

Accelerated solvent extractor (ASE 100, Dionex, Sunnyvale, CA, USA) was used for the extraction of a dried and powdered root of *Berberis vulgaris* that were purchased in a local herbal shop in Lublin, Poland. The plant material was produced by Proherbis company (Proherbis Jaroslaw Wolanski, Debiec, Poland). The Extraction was performed on the portions of 25 g of powdered plant material in a large – 66 mL – extracting cell in the following conditions: temperature of extraction: 80 ◦C, static time: 5 min, number of cycles: 4, purge time: 120 s, purge volume: 120 %. The obtained extracts were joined and evaporated to dryness on a rotary evaporator at 45 ◦C. The dried residue was used for the isolation of MAG on Armen SCPC-250-L centrifugal partition chromatograph (Brittany, France) equipped in a 250 mL column, a quaternary pump, a UV detector and a fraction collector. Before the separation the composition of a biphasic solvent system was performed revealing the mixture of chloroform: methanol: water in the ratio 4: 3: 3 (*v*/*v*/*v*) as the most selective one for the recovery of MAG separately from the remaining isoquinoline alkaloids. The addition of 20 mM of hydrochloric acid to the upper phase and 20 mM of triethylamine to the lower phase enhanced the separation efficiency. The fractionation was performed on 500 mg of extract that was initially dissolved in 5 mL of unequally volumed mixture of the upper and the lower phases (with the addition of trietylamine with o hydrochloric acid). First the acidified stationary phase was pumped into the column (at 500 rpm and 20 mL/min) and the sample was injected together with the basified mobile phase at the rotation speed of 1300 rpm and the flow rate of 6 ml/ min. After 50 min extrusion of the column content was performed. Then, MAG was eluted from the column at 64th min of fractionation. Its purity was assessed by an HPLC-ESI-QTOF-MS/MS platform (Agilent Technologies, Santa Clara, CA, USA) in the same method as described in the manuscript in the section on HPLC-MS analysis in the former manuscript of the authors[16].

Figure S1

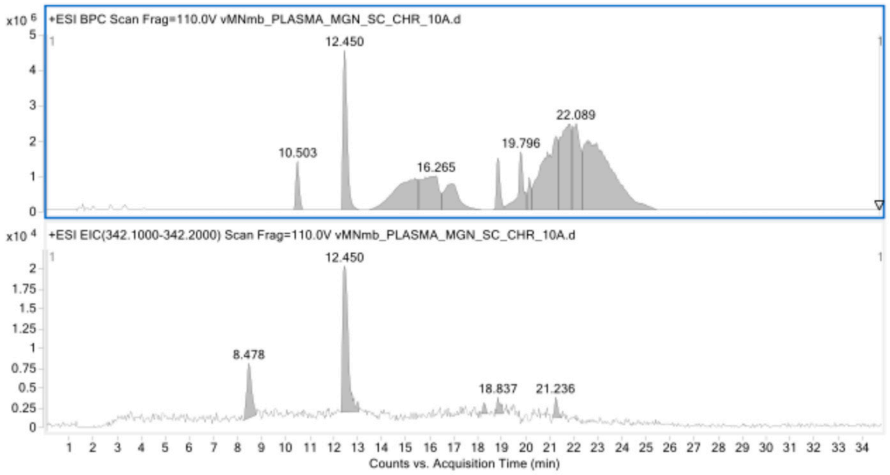

Figure S1. A sample total ion mass chromatogram of the plasma sample (above) and an extracted ion chromatogram of magnoflorine (MAG) (below) with the alkaloid at 12.4 min

Figure S2

Entity Name	Compound Name	vMNmb_PLASMA_SOL_...	v...	vMNmb_PLASMA_MGN_SC_CHR_10...	.
Phosphatidylcholines	PC(14:1(9Z)/14:1(9Z))	0	0	16.72	

.	vMNmb_PLASMA_MGN_SC_CHR_20...	...	DB	DB ID	Compound	Retention Time	Formula
	17.043		KEGG ID	C00157	PC(14:1(9Z)/14...	12.435	C36 H69 N O8 P

Figure S2. The details of the chemometric analysis of the samples showing in the Kegg Pathways analysis the details obtained for plasma sample of control and two treated groups with 10 mg/kg and 20 mg/ kg MAG
